# Typical radiological progression and clinical features of patients with coronavirus disease 2019

**DOI:** 10.18632/aging.103170

**Published:** 2020-05-02

**Authors:** Min Wang, Linghong Guo, Qi Chen, Guojin Xia, Bo Wang

**Affiliations:** 1 Department of Radiology, The First Affiliated Hospital of Nanchang University, Nanchang 330006, Jiangxi Province, China

**Keywords:** COVID-19, 2019 novel coronavirus pneumonia, radiological features, chest CT, ground-glass opacity

## Abstract

We aimed to describe typical radiological features and progression of Coronavirus disease 2019 (COVID-19) patients. We reviewed the chest CT scans, laboratory findings, and clinical records of 66 COVID-19 patients who were admitted to affiliated hospitals of Nanchang university, Nanchang, China, from Jan 21 to Feb 2, 2020. CT was used to evaluate the radiological characteristics of COVID-19 patients. Only 4 patients (4/66, 6%) claimed their exposure to COVID-19 pneumonia patients. The major symptoms were fever (60/66, 91%) and cough (37/66, 56%). The predominant features of lesion were scattered (43/66, 65%), bilateral (50/66, 76%), ground-glass opacity (64/66, 97%), and air bronchogram sign (47/66, 71%). Forty-eight patients (48/66, 73%) had more than two lobes involved. Right lower lobe (58/66, 88%) and left lower lobe (49/66, 74%) were most likely invaded. Twelve patients (12/66, 18%) had at least one comorbid condition. Pleural traction (29/66, 44%), crazy paving (15/66, 23%), interlobular septal thickening (11/66, 17%), and consolidation (7/66, 11%) were also observed. The typical radiology features of COVID-19 patients are scattered ground-glass opacity in the bilateral lobes. Fever and cough are the major symptoms. Evaluating chest CT, clinical symptoms, and laboratory results could facilitate the early diagnosis of COVID-19, and judge disease progression.

## INTRODUCTION

Since December 2019, a series of unknown pneumonia caused by a novel coronavirus broke out in Wuhan, Hubei, China. This new coronavirus was named as severe acute respiratory syndrome coronavirus 2 (SARS-Cov-2) or 2019 novel coronavirus (2019-nCoV) [1]. The disease caused by 2019-nCoV is coronavirus disease 2019 (COVID-19), which had been confirmed to be a global pandemic by the World health organization (WHO). By April 8 2020, more than 1, 350, 000 infected cases and 79, 000 deaths have been caused by COVID-19 [2]. COVID-19 has been effectively prevented and controlled in China, Singapore, South Korea, and Japan right now, but 2019-nCoV is spreading fast in Europe and the United State. Obviously, the threat to the global health and economy by 2019-nCoV will last for a long time [3, 4].

2019-nCoV, a betacoronavirus, is a member of family Coronaviridae [5]. In total, six types of coronavirus have been identified including middle east respiratory syndrome coronavirus (MERS-CoV), severe acute respiratory syndrome coronavirus (SARS-CoV), NL-63, OC-43, and 229E, among which MERS-CoV and SARS-CoV could cause severe respiratory diseases [6]. 2019-nCoV, a novel coronavirus, could interact with the human angiotensin converting enzyme 2 receptor through its spike protein [7, 8]. 2019-nCoV spread among population mainly through respiratory droplets and direct contact, and could cause several different symptoms including fever, cough, and fatigue [9].

Early diagnose of 2019-nCoV is important for the next isolation and treatment. However, shortage of nucleic acid detection reagent has been reported in some countries. CT characterized by convenience and accuracy plays a key role in the diagnose of respiratory diseases. CT provides a simple, direct, and convenient auxiliary diagnosis method for the patients, who cannot be tested by RT-PCR. However, there are very few studies focusing on the lung CT features of COVID-19 patients so far.

In this study, we summarized the radiological characteristics and clinical features of 66 COVID-19 patients. We aimed to unfold the typical radiology characteristics and progression of COVID-19 patients. This study may provide helpful images for early diagnose and treatment.

## RESULTS

Total 66 COVID-19 pneumonia patients were admitted to three affiliated hospitals of Nanchang university between Jan 21 to Feb 2, 2020 (Table 1). Epidemiological investigation indicated that 4 (4/66, 6%) patients had direct exposure to COVID-19 pneumonia patients, 21 (21/66, 32%) patients lived in or visited Wuhan during epidemic, and 41 (41/66, 62%) patients did not have obvious exposure. In this cohort, the average age of all patients was 44 years (SD 14; range 18-75), and there were 43 male patients (43/66, 65%) and 23 female patients (23/66, 35%). 45 (45/66, 68%) patients were younger than 50 years, and 21 (21/66, 32%) patients were elder than 50 years.

**Table 1:** Clinical characteristics and laboratory results of patient with COVID-19 pneumonia (n=66).

**Characteristic**	**Number (%)**
Male	43 (65%)
Female	23 (35%)
> 50 years old	21 (32%)
≤ 50 years old	45 (68%)
Exposure to COVID-19 pneumonia patients	4 (6%)
Lived in or visited Wuhan during the epidemic	21 (32%)
Unknown exposure	41 (62%)
Symptoms	
Fever	60 (91%)
Cough	37 (56%)
Sore throat	17 (26%)
Sputum	16 (24%)
Fatigue	15 (23%)
Dyspnoea	14 (21%)
Dizziness	9 (14%)
Myalgia	7 (11%)
Headache	3 (5%)
Diarrhoea	3 (5%)
Nausea	3 (5%)
Rhinorrhea	2 (3%)
C-reactive protein (mg/L; normal range 0-10)	
Increased	38 (58%)
Decreased	0
Normal	28 (42%)
Leucocytes (× 10^9^, normal range 3.5-9.5)	
Increased	1 (2%)
Decreased	14 (21%)
Normal	51 (77%)
Lymphocyte (× 10^9^, normal range 1.1-3.2)	
Increased	0
Decreased	29 (44%)
Normal	37 (56%)
Comorbid conditions	
Any	12 (18%)
Hepatitis or liver cirrhosis	8 (12%)
Hypertension	4 (6%)
Diabetes	2 (3%)
Chronic pulmonary disease	1 (2%)
Cardiovascular disease	1 (2%)

The most common symptoms were fever (60/66, 91%) and cough (37/66, 56%). Some other symptoms, such as sore throat (17/66, 26%), sputum (17/66, 24%), fatigue (15/66, 23%), and dyspnoea (14/66, 21%), were also observed frequently. Other non-specific symptoms included myalgia (7/66, 11%), headache (3/66, 5%), diarrhea (3/66, 5%), and nausea (3/66, 5%). 38 (38/66, 58%) patients had a higher level of C-reactive protein, and the rest of patients were normal. The leucocytes level of 51 (51/66, 77%) patients was normal. 29 (29/66, 44%) patients had decreased lymphocyte level, and 37 (37/66, 56%) patients’ lymphocyte count was normal. Comorbid conditions were not common in these patients, and only 12 (12/66, 18%) patients had at least one complication.

Scattered lesions found in 43 (43/66, 65%) patients were most common, and 23 (23/66, 35%) patients shown subpleural distribution. Lesion involved bilateral lungs was observed in 50 (50/66, 76%) patients. Lesion invaded more than two lobes was found in 48 (48/66, 71%) patients. Right lower lobe (58/66, 88%) and left lower lobe (49/66, 74%) were most likely to be involved.

**Figure 1:**
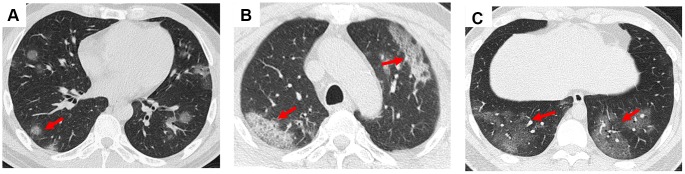
**Ground-glass opacity and crazy paving in the CT scans of COVID-19 pneumonia patients.** (**A**) Multiple nodular ground-glass opacity scattered in both lungs of a 44-year-old male patient; (**B**) Mixed ground-glass opacity along the long axis of subpleural in both lungs of a 67-year-old male patient; (**C**) Crazy paving was observed in the bilateral lower lungs of a 67-year-old male patient at the fourth day since admission. Typical lesions were marked with red arrows.

**Figure 2:**
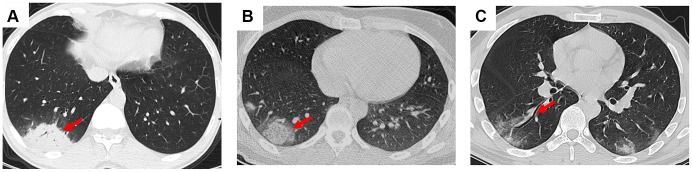
**Consolidation, halo sign, and bronchiectasia in the CT scans of COVID-19 pneumonia patients.** (**A**) Consolidation accompanying air bronchogram sign was found in the right lower lobe of a 46-year-old male patient; (**B**) Halo sign was observed in the right lower lobe of a 18-year-old male patient; (**C**) Bronchiectasia was observed in the right lower lobe of a 30-year-old male patient with bilateral ground-glass opacity. Typical lesions were marked with red arrows.

In this study, we presented some common and typical radiology changes (Figures 1 and 2). The most common radiology characteristic seen on the CT was ground-glass opacity (64/66, 97%). Most ground-glass opacities were characterized by scattered and bilateral lesions (Figure 1A and 1B). The CT scans of 15 (15/66, 23%) patients shown crazy paving (Figure 1C), and consolidation was observed in 7 (7/66, 11%) patients (Figure 2A). In addition, air bronchogram sign (47/66, 71%, Figure 1D), pleural traction (29/66, 44%), interlobular septal thickening (11/66, 17%), and halo sign (3/66, 5%, Figure 2B) were also observed (Table 2). Bronchiectasia was observed in the right lower lobe of one patient with bilateral ground-glass opacity (Figure 2C).

**Table 2:** CT findings of patient with COVID-19 pneumonia (n=66)

**CT features**	**Number (%)**
Distribution	
Subpleural	23 (35%)
Central	0
Scattered	43 (65%)
Number of lobes involved	
1	13 (20%)
2	5 (8%)
3	10 (15%)
4	11 (17%)
5	27 (41%)
More than two lobes involved	48 (73%)
Lobe of lesion distribution	
Right upper lobe	42 (64%)
Right middle lobe	37 (56%)
Right lower lobe	58 (88%)
Left upper lobe	44 (67%)
Left lower lobe	49 (74%)
Lesion involved bilateral lungs	50 (76%)
Lesion involved unilateral lung	16 (24%)
Lesion characteristics	
Ground-glass opacity	64 (97%)
Crazy paving	15 (23%)
Consolidation	7 (11%)
Lesion shape	
Patch	66 (100%)
Circular	13 (20%)
Reticular spline	12 (18%)
Other signs in the lesion	
Air bronchogram sign	47 (71%)
Pleural traction	29 (44%)
Interlobular septal thickening	11 (17%)
Vacuole Halo sign	3 (5%)
Other findings	
Pulmonary emphysema	4 (6%)
Pulmonary fibrosis	2 (3%)
Pleural effusion	1 (2%)
Bronchiectasis	1 (2%)
Tuberculosis	1 (2%)

**Figure 3:**
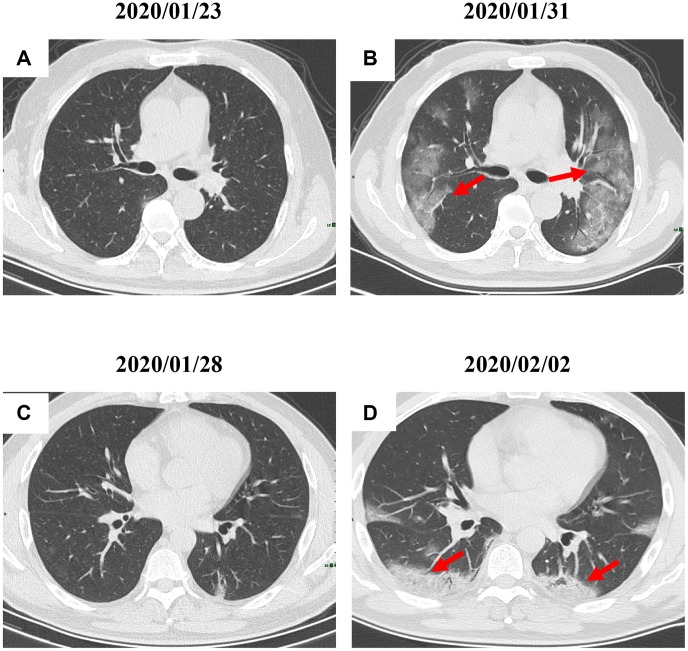
**Radiological worsen progression of two COVID-19 pneumonia patients.** (**A**, **B**): Bilateral, large, and multiple ground-glass opacity was observed in a 47-year-old male patient with type 2 diabetes after 8 days since admission; (**C**, **D**) Consolidation accompanying air bronchogram were found in the bilateral lower lungs of a 29-year-old male patient after 5 days since admission. Typical lesions were marked with red arrows.

**Figure 4:**
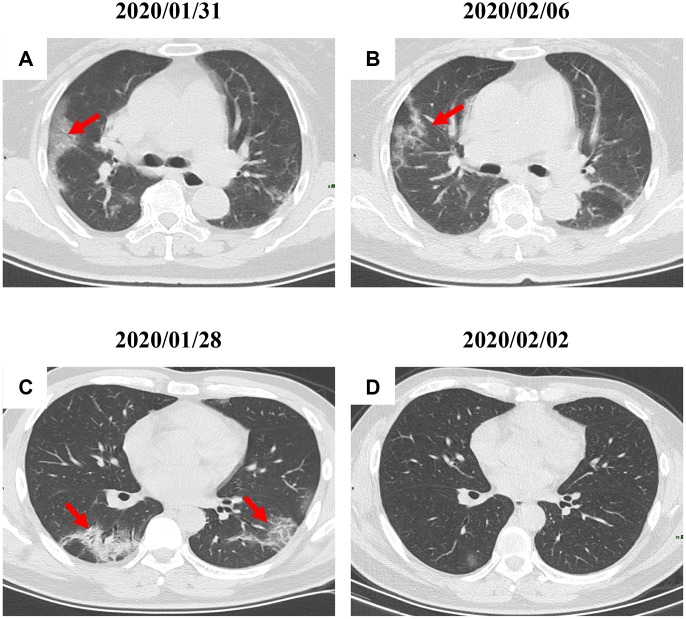
**Radiological improvement of two COVID-19 pneumonia patients.** (**A**, **B**) Patch lesions were absorbed and changed into reticular spline ones (a 31-year-old female patient); (**C**–**D**) Significant improvement of CT sign was achieved in a 22-year-old male patient. Typical lesions were marked with red arrows.

**Figure 5:**
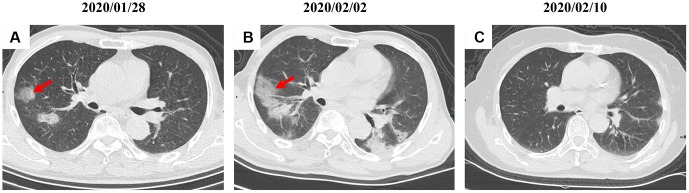
**A serial CT images after admission of a 54-year-old male patient.** (**A**) Patch ground-glass opacity was observed in the middle right lobe. (**B**) 5 days later, significant larger patch ground-glass opacities were observed in bilateral lungs. (**C**) Follow-up CT scans on day 13 after admission show a remarkable improvement. Typical lesions were marked with red arrows.

**Figure 6:**
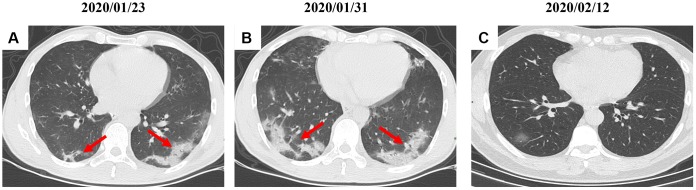
**A serial CT images after admission of a 54-year-old female patient.** (**A**) Patch ground-glass opacity mainly located in the left lower lobe. (**B**) Significant larger patch ground-glass opacities were observed in both lower lobes after 8 days. (**C**) Follow-up CT scans on day 20 after admission show a remarkable improvement. Typical lesions were marked with red arrows.

By Mar 23, 2020, 60 (60/66, 91%) patients had been discharged. 6 (6/66, 9%) patients were still in hospital, and two patients had died because of ARDS. Patient 1, 78-year-old man with hypertension, who died on day 15 after admission (Figure 1B). Patient 2, 47-year-old man with type 2 diabetes, whose CT scan presented rapid radiology progression (Figure 3A, 3B). The radiological change of COVID-19 pneumonia develops fast during the first seven days (Figure 3C, 3D). Some of patch lesion could be absorbed and change into reticular spline lesion (Figure 4A, 4B). Meanwhile, some patients achieved rapid recovery with significant improvement of CT sign (Figure 4C, 4D) and clinical symptoms. We also did some CT follow-up scans for few patients, which showed the aggravated progression of disease since admission and rapid recovery after treatment (Figures 5 and 6). Disappearance of lesions and significant improvement of clinical symptoms were observed in two patients (Figure 5: a 54-year-old male patient; Figure 6: a 54-year-old female patient).

## DISCUSSION

2019-nCoV, an enveloped positive-sense RNA virus, is the seventh member of the coronaviridae family [10]. It is estimated that 2019-nCoV could cause 1%-6% mortality rate depending on different regions, which is lower than MERS-CoV (10%) and SARS-CoV (37%) [5, 11], but the high infectivity of the pathogen has caused a global pandemic. 2019-nCoV has became a huge threat for the global health, economic development, and social stability.

Previous study indicated that old age population with comorbidities were susceptible to infection of 2019-nCoV [12]. In our cohort, there were 45 (45/66, 68%) patients under 50 years-old, and only 12 (12/66, 8%) patients had at least one comorbid condition. Small cohorts and differences in demographic characteristics might account for this discrepancy. Previous study suggested that 73% (30/41) patients were male [1], which is inconsistent with another study [13]. In our study, male infected patients account for 65% (43/66). The difference of gender distribution might also due to small cohorts. By Mar 23, 2020, two patients (78 and 47 years old, respectively) in this study had died, and both had comorbid conditions.

It is worth mentioning that 41 patients (41/66, 62%) patients had no obvious exposure history indicating that they might be infected by latent infection patients. Latent infection should attract the attention of people, because the clinical appearance of latent infection patients is not consistent with real disease progression. Meanwhile, the latent infection patients indeed have infectivity. When participating in group activities or gathering, wearing mask should be an effective method to prevent infectivity by asymptomatic patients. Some countries such as India and Indonesia have a large population and the medical condition of them is not optimistic. For the people who lack sufficient medical protection, it is effective to prevent and control virus spreading by avoiding gathering, wearing mask in the crowd, regular ventilation at home.

Fever and cough were the most common symptoms in the COVID-19 patients. Self-isolation and wearing mask are still effective and economic method for fever people who have mild symptoms, but if symptoms aggravate, professional and medical measures should be taken because of the high mortality rate.

Due to special structure, right lower lobe and left lower lobe were most commonly involved, which is in line with previous study [13]. Most COVID-19 patients presented bilateral lungs lesion with scattered distribution. However, unilateral lesion is more common in the early infection stage of MERS-CoV and SARS-CoV [14, 15]. The most common image feature was ground-glass opacity, which was found in 64 (64/66, 97%) patients. Other features such as crazy paving, consolidation, air bronchogram sign, and pleural traction were also observed. However, these radiological characteristics could be found in other viral pneumonia caused by MERS-CoV, SARS-CoV, and adenovirus.

RT-PCR has been viewed as the gold standard for COVID-19 pneumonia diagnosis. While, many countries are facing the shortage of nucleic acid test reagent. Meanwhile, the it nucleic acid test costs at least 4-5 hours including throat swab collection, RNA extraction, and RT-PCR. Chest CT could provide effective and fast evidence for the clinical diagnosis of COVID-19 pneumonia. Imaging findings could also indicate the prognosis. The radiological features of some patients might worsen fast indicating a poor prognosis (Figure 3A, 3B).

Our study had some limitations. Due to short time for data collection, we did not conduct long-term follow-up CT, which is necessary to evaluate the prognosis of patients. In addition, we did not systematically investigate the radiology progression of patients, which could help to judge disease course of COVID-19 pneumonia.

In summary, the typical radiology features of COVID-19 pneumonia were characterized by bilateral and scattered ground-glass opacity accompanying with air bronchogram sign, and predominant lesion location in the left lower lobe and right lobe. Sometimes, the clinical symptoms were not consistent with imaging features indicating that asymptomatic patients may account for a certain proportion. Therefore, CT should be an effective, fast, and simple method for the screening, diagnose, and treatment of COVID-19 pneumonia.

## MATERIALS AND METHODS

### Patients

The retrospective study was approved by the ethical committee of affiliated hospitals of Nanchang university. The written informed consent of this research has been waived by the ethics committee of our hospital for the reason that there is no potential risk and this is a retrospective study. The COVID-19 patients identified by RT-PCR or nest-generation sequencing were admitted from Jan 21 to Feb 2, 2020. A total of 66 patients were enrolled (43 men and 23 women, 18-75 years old, average age: 44 years). Throat swab samples were collected by experienced nurses, and total RNA extraction was conducted using TRIzol reagent (Thermo scientific, CA, USA). According to previous study [13], related primers (forward primer: 5′-TCAGAATGCCAATCTCCCCAAC-3′; reverse primer: 5′-AAAGGTCCACCCGATACATTGA-3′) were used to detect SARS-CoV-2.

### CT data acquisition

All patients were examined by CT for 2-6 times at different time points. The patients in the supine position were scanned using Siemens Emotion 16 (Siemens Healthineers, Forchheim, Germany), Phillips iCT 256 (Phillips Healthcare, Andover, MA, USA), or GE revolution frontier (GE Healthcare, Issaquah, WA, USA). Scans were conducted from the apex of lung to the base of lung on the condition that patients were instructed to hold breath during examination. The following scan parameters were used: tube voltage 120 kV, tube current 70-168mAs, pitch 08-1.2 mm, slice thickness 5 mm, matrix 512×512, FOV 55*35cm, axial reconstruction image layer thickness 1-1.5mm. Three experienced radiologists blinded to nucleic acid results of patients, reviewed all CT scans.

### Data analysis

Data analysis was performed on SPSS 22.0 (IBM, Armonk, NY, USA). Categorical variables were presented as number (%), and continuous variables were shown as a range.
